# Association between higher mortgage payment-to-income ratio and greater psychological distress among high-income homeowners in Japan: A cross-sectional study

**DOI:** 10.1016/j.pmedr.2025.102987

**Published:** 2025-01-23

**Authors:** Kazuya Ogawa, Keiichi Shimatani, Ryotaro Iwayama, Norimichi Suzuki

**Affiliations:** a Center for Preventive Medical Sciences, Chiba University, 1-33 Yayoi-cho, Inage-ku, Chiba 263-8522, Japan; b Graduate School of Medical and Pharmaceutical Sciences, Chiba University, 1-8-1 Inohana, Chuo-ku, Chiba 260-8670, Japan; c Comprehensive Housing R&D Institute, Sekisui House, Ltd., 6-6-4 Kabutodai, Kizugawa, Kyoto 619-0224, Japan

**Keywords:** Mental Health, Psychological distress, Mortgage, Housing affordability, Housing tenure, Japan

## Abstract

**Objective:**

While the relationship between higher housing cost burden and poorer mental health has been established in lower-income groups, research examining high-income populations is scarce. We aimed to examine this relationship among high-income homeowners in Japan.

**Methods:**

We conducted a cross-sectional study on 6753 Japanese homeowners in detached houses. Data were collected as part of an ongoing panel survey, from new survey respondents during Wave 2 (Jul-Sept 2023) and Wave 3 (Jan-Mar 2024). Psychological distress, measured using the Kessler 6 scale, was the dependent variable. The explanatory variable was the mortgage-to-income ratio, categorized into two and six levels. We performed modified log Poisson regression analyses, incorporating income stratification, with missing data handled by multiple imputation.

**Results:**

For both overall respondents (PR = 1.22, 95 % CI 1.11–1.35) and the high-income group (PR = 1.23, 95 % CI 1.09–1.39), individuals with mortgage payment ratios ≥30 % were more likely to report psychological distress compared with those <30 %. While the low-income group showed a marginally similar tendency (PR = 1.21, 95 % CI 1.00–1.46), this association was not robust in the sensitivity analysis.

**Conclusion:**

Higher mortgage payment ratios were associated with greater psychological distress even among high-income homeowners. The impact of housing cost stress on psychological distress may be more extensive than previously recognized.

## Highlights

• High mortgage costs were associated with greater psychological distress among high earners.
• Mortgage payments and distress had a non-linear relationship with two thresholds.
• High housing costs pose mental health risks in various socioeconomic groups.

## 1 Introduction

Housing costs represent the largest lifetime expenditure for many people and constitute an ongoing financial burden. Compared with other expense categories (e.g., food costs), reducing housing expenses is challenging. Consequently, housing costs can be a source of significant psychological stress.

### 1.1 Literature review

Research on the relationship between higher housing cost burden and poorer mental health has primarily been conducted in Australia and South Korea, utilizing standardized measurement scales such as the Mental Component Summary (MCS) and the Center for Epidemiologic Studies Depression Scale (CES—D) (Bentley et al., 2011, Bentley et al., 2012; Bentley et al., 2016a; Bentley et al., 2016b; Baker et al., 2020a; Baker et al., 2020b; Bentley et al., 2022; Mason et al., 2013; Singh et al., 2020; Botha et al., 2024; Park and Jung, 2019; Park and Seo, 2020; Park and Seo, 2022; Park et al., 2023; Kim et al., 2021).

These studies demonstrated the association between housing cost burden and mental health, varying by demographic and housing factors. Bentley et al. (2012) reported this association among women. Age-related variations in this association were observed across different age groups, with both older adults (≥60 or ≥ 65 years) and young adults (25–34 years) showing adverse effects (Bentley et al., 2022; Park and Seo, 2022; Arundel et al., 2024). Multiple studies showed this association, with stronger effects among renters compared to homeowners (Mason et al., 2013; Park and Jung, 2019; Kim et al., 2021; Botha et al., 2024; Arundel et al., 2024).

There are methodological limitations in evaluating this association among high-income groups. Most existing studies define “housing affordability stress” using the following criteria: 1) housing costs comprising ≥30 % of gross household income, and 2) household income falling within the bottom 40 % of the national income distribution.

Under this definition, high-income individuals who spend a large portion of their income on housing are excluded from the assessment of housing affordability stress. Following Bentley et al.'s (2011) findings of no significant associations in high-income groups, subsequent studies have predominantly examined this association in low-income populations (Bentley et al., 2012; Bentley et al., 2016a; Kavanagh et al., 2016; Singh et al., 2020; Arundel et al., 2024; Botha et al., 2024). Existing research has assumed that high-income groups are unlikely to experience housing cost burden severe enough to impact mental health.

Housing costs are primarily classified into two types: rent paid by tenants and mortgages payments by homeowners. Mortgages represent a form of debt, and concerns about being able to repay borrowed funds may cause a source of stress (Drentea and Reynolds, 2012; Hamilton et al., 2019). Researchers in the United States (Bushman and Mehdipanah, 2022) and Canada (Cairney and Boyle, 2004) have shown that homeowners with mortgages exhibit poorer mental health compared to those without mortgages. Furthermore, Canadian research has reported a higher food insecurity risk among residents with mortgages (Fafard St-Germain and Tarasuk, 2020).

Population-specific differences have also been reported. A study across three European countries found that mortgages ≥20,000 euros were associated with depressive symptoms only among women (Hiilamo and Grundy, 2020). However, research on the impact of mortgages by income groups remains limited. Mason et al. (2013) demonstrated that mental health scores were lower among homeowners in the top 50 % income bracket with mortgage payment ratios ≥30 % compared to those with ratios <30 %. However, this study did not analyze high-income groups in detail as it was not the main focus.

### 1.2 Mortgage burden and psychological distress in Japan

In Japan, the relationship between mortgage debt and psychological distress merits particular examination. According to the Ministry of Health, Labour and Welfare (2022), 29 % of the Japanese population exhibits psychological distress (Kessler Psychological Distress Scale (K6) score ≥ 5). Among working families of two or more people in Japan, 82 % are homeowners, with 53 % holding mortgage loans. (Ministry of Internal Affairs and Communications, 2023). Of these mortgage holders, 20 % have a mortgage payment ratio ≥ 30 % (Japan Housing Finance Agency, 2023). Purchasers of new detached houses earn 1.5 times the average household income; however, 65 % of these mortgage holders report experiencing financial strain from their loan payments (Ministry of Land, Infrastructure, Transport and Tourism, 2022). These data indicate that high-income homeowners experience significant financial strain due to these obligations. Research focusing on this unique Japanese context holds important implications for understanding the impact of mortgage payment on psychological distress among high-income homeowners.

### 1.3 Hypothesis

Considering the Japanese data, we hypothesize that the higher mortgage payment ratio is associated with greater psychological distress, even among high-income homeowners. This hypothesis has both practical and academic significance. Practically, our findings may have implications for government agencies to develop targeted support programs and guidelines for homeowners across all income levels, including high-income households, an often overlooked demographic in terms of psychological vulnerability. In Japan, this population shows lower treatment-seeking behaviors compared with low-income individuals due to occupational stigma and concerns about income loss from work absence, potentially leading to more severe outcomes (Fukuda and Hiyoshi, 2012). Academically, it contributes to the growing research on housing and psychological distress. While Tomioka et al. (2019) found that homeowners generally exhibit better health than renters, the specific impact of mortgage payment ratios on homeowners' psychological distress, particularly among high-income individuals, remains understudied. The current study aimed to examine the correlation between higher mortgage payment ratios and greater psychological distress among high-income homeowners.

## 2 Methods

### 2.1 Data

We performed a cross-sectional analysis using data from the Japan Housing and Health Cohort (J-Hohec). J-Hohec was established to elucidate the association between living environments and residents' health status and apply these findings to future housing design. J-Hohec consists of objective floor plan data from houses built by a major Japanese housing company, along with survey data regarding homeowners' socioeconomic status and health conditions. J-Hohec is an ongoing panel survey, with three waves of data collection completed as of August 2024.

The housing company's voluntary Owners Club for home construction contract holders had approximately 470,000 members as of April 2024. Web-based survey invitations were sent to approximately 270,000 members who had agreed to receive social media and email communications. One household member participated voluntarily after providing consent through the web-based questionnaire.

The five-week survey included three reminder notifications and followed consistent recruitment procedures throughout the waves. The response rate for waves two and three, which are the focus of this study, was approximately 5 %. Due to selective sampling (limited to members accepting social media/email communications) and low response rate, results may not represent the full Owners Club membership.

The dataset construction and participant selection process was as follows. The initial sample size was 7947 individuals. This included new survey respondents from Wave 2 (conducted from July to September, 2023, *n* = 4250) and Wave 3 (conducted from January to March 2024, *n* = 3697), which contained the primary explanatory variable of this study (the mortgage payment ratio). From this initial sample, we excluded apartment-type housing owners because it included data from rental apartment owners (*n* = 789) and respondents with a history of sleep disorders or mental health issues (*n* = 405) to mitigate reverse causality bias. The final analysis included 6753 respondents. Missing data (ranging from 0 % to 16 %) were handled using multiple imputation.

The dataset contains key variables necessary for analyzing the relationship between mortgage ratios and psychological distress, with, 2023–2024 data reflecting contemporary living conditions that make it well-suited for our analysis.

### 2.2 Outcome variables

The outcome variable in this study was psychological distress, as measured using the K6 scale (Kessler et al., 2002). The K6 consists of six items asking about experiences in the past 30 days (nervous, hopeless, restless or fidgety, depressed, everything was an effort, and worthless), each rated on a five-point scale from 0 to 4. The total score across the six items indicates the severity of depressive and anxiety symptoms. Higher scores reflect greater severity of psychological distress. Based on the method described by Sakurai et al. (2011), we set the cut-off point at 5. K6 scores of ≥5 were classified as indicating psychological distress, whereas scores <5 were considered to indicate no psychological distress.

### 2.3 Exposure variables

The explanatory variable used was the ratio of mortgage payments to gross household income. Respondents were asked about “the proportion of monthly household income spent on mortgage (excluding utilities).” Responses were collected on a six-point scale: “None,” “<10 %,” “About 10 %,” “About 20 %,” “About 30 %,” and “≥40 %.” For statistical analysis, two types of variables were used. The first, based on prior research by Mason et al. (2013), categorized the mortgage payment ratio into two groups: <30 % and ≥ 30 %. The second used the original six-point scale as is.

### 2.4 Control variables

This study used individual, housing, neighborhood factors, and survey timing as control variables. Individual factors included age (categorized as under 45, 45–64, and ≥ 65 years), gender (male, female, or do not want to answer), education level (less than a university degree and a university degree or higher), household income (<4 million JPY (27.5 K USD), 4–6 million JPY (27.5 K–41.5 K USD), 6–8 million JPY (41.5 K–55.0 K USD), and ≥ 8 million JPY (55.0 K USD)), household size (single-person and multi-person), exercise and drinking habits (<3–4 times per week and ≥ 3–4 times per week), smoking status (smoker and non-smoker), and the occurrence of significant life events in the past year (yes, no, and do not want to answer). Significant life events included the death of a close relative or friend, separation, divorce, serious illness (of oneself or family/friends), disasters, and unemployment. Housing factors considered total floor area, categorized into quartiles: very small (Q1), small (Q2), large (Q3), very large (Q4). Neighborhood factors included the presence of natural environments around home and population density of habitable areas. Natural environments, categorized as present and absent, were assessed in response to the question, “Do you experience natural environments (such as green spaces or water features) in your neighborhood?” Population density was categorized into quartiles: very low (Q1), low (Q2), high (Q3), very high (Q4).

### 2.5 Statistical analysis

Our analyses proceeded in four steps. First, we calculated descriptive statistics for the sample overall and by income. Initial descriptive statistics were computed using the pre-imputation dataset, with all subsequent analyses performed using multiply imputed data. Second, we examined the bivariate relationship between high mortgage ratios and psychological distress, stratified by income. Based on Ministry of Health Labour and Welfare (2023), which showed Japan's median and mean household incomes at 4.05 and 5.24 million JPY respectively, the first measurable income cut-off above these values was 6 million JPY (41.5 K USD). The same national household survey showed this value represented the top 30 % of Japan's income distribution. We therefore classified households as low-income (<6 million JPY) or high-income (≥6 million JPY). Third, we replaced the binary mortgage payment ratio with a six-category variable to gain more nuanced insights. Fourth, a sensitivity analysis using data from homeowners under 65 was conducted to avoid overestimation of mortgage-to-income ratios among retirement-age homeowners, who typically have lower incomes due to retirement, leading to their potential overrepresentation in lower-income categories with higher ratio values.

To handle missing data (0 %–16 %), we implemented multiple imputation using Amelia (Honaker et al., 2011). This method was chosen as it improves accuracy and statistical power compared to other missing data techniques. We created 20 imputed datasets incorporating all analytical variables used in this study. We then conducted the regression analyses on each imputed dataset and pooled the results according to Rubin's rules. We adopted modified log Poisson regression (Poisson regression with a robust error variance) for analyzing our binary outcome variable, following recent recommendations (Gallis and Turner, 2019; Gnardellis et al., 2022). Standard Poisson regression applied to binary data tends to overestimate standard errors of prevalence ratios (PR) (Zou, 2004); thus, modified log Poisson regression resolves this limitation through robust error estimation. While logistic regression approximates PR using odds ratios (OR), this approximation can be inaccurate when the outcome incidence exceeds 10 % (Gnardellis et al., 2022). Because the outcome incidence in the current study was 29 %, approximating PR using OR would have been inappropriate. We used R version 4.3.3 (R Core Team, 2024) with the stats and sandwich packages.

### 2.6 Ethical approval

This study was conducted with the approval of The Research Ethics Committee of the Graduate School of Medicine, Chiba University (approval number: M10381) and in accordance with the principles of the Declaration of Helsinki.

## 3 Results

### 3.1 Demographic characteristics

Table 1 shows the characteristics of respondents before multiple imputation. They had higher socioeconomic status than the general Japanese population. The average age was 53.8 years, with 74 % under 65. Males comprised 72 % of respondents, likely reflecting the predominant male registration in the Owners Club. The respondents' university degree attainment (66 %) exceeded the national average (22 %) (Ministry of Internal Affairs and Communications, 2020). High-income earners (annual income ≥6 million JPY) constituted 66 % of the sample, far above Japan's national rate of 30 % (Ministry of Health, Labour and Welfare, 2023). The high-income group was younger (90 % vs 49 % under 65) and more educated (72 % vs 58 % with university degrees or higher) compared to the low-income group.

**Table 1:** Descriptive statistics of homeowners, overall sample and by income groups in the Japan Housing and Health Cohort, 2023–2024.

**Characteristics**	**Overall, N (%)** *N* = 6753	**Low, N (%)** *N* = 1949	**High, N (%)** *N* = 3709	**NA, N (%)** *N* = 1095	**p-value**^1^
**Psychological distress**					0.974
No	4768 (71)	1373 (70)	2623 (71)	772 (71)	
Yes	1985 (29)	576 (30)	1086 (29)	323 (29)	
NA	0	0	0	0	
**Mortgage payment ratio**					<0.001
Less than 30 %	5299 (85)	1620 (87)	2928 (83)	751 (93)	
30 % or more	916 (15)	250 (13)	606 (17)	60 (7)	
NA	538	79	175	284	
**Age**					<0.001
Under 45	1978 (29)	331 (17)	1482 (40)	165 (15)	
45–64	3047 (45)	623 (32)	1849 (50)	575 (53)	
65 or older	1728 (26)	995 (51)	378 (10)	355 (32)	
NA	0	0	0	0	
**Gender**					<0.001
Male	4841 (72)	1462 (75)	2719 (73)	660 (60)	
Female	1812 (27)	469 (24)	967 (26)	376 (34)	
Do not want to answer	100 (1)	18 (1)	23 (1)	59 (5)	
NA	0	0	0	0	
**Educational attainment**					<0.001
Less than university	2321 (34)	820 (42)	1049 (28)	452 (41)	
University or higher	4432 (66)	1129 (58)	2660 (72)	643 (59)	
NA	0	0	0	0	
**Household size**					<0.001
Single-person	391 (6.0)	149 (8.2)	176 (4.8)	66 (6.3)	
Multi-person	6111 (94)	1667 (92)	3466 (95)	978 (94)	
NA	251	133	67	51	
**Exercise habits**					<0.001
Low frequency	4598 (68)	1109 (57)	2759 (74)	730 (67)	
High frequency	2155 (32)	840 (43)	950 (26)	365 (33)	
NA	0	0	0	0	
**Drinking habits**					<0.001
Low frequency	4935 (73)	1348 (69)	2789 (75)	798 (73)	
High frequency	1818 (27)	601 (31)	920 (25)	297 (27)	
NA	0	0	0	0	
**Smoking status**					0.010
Non-smoker	6038 (89)	1767 (91)	3278 (88)	993 (91)	
Smoker	715 (11)	182 (9)	431 (12)	102 (9)	
NA	0	0	0	0	
**Significant life events**					<0.001
No	5071 (75)	1384 (71)	2900 (78)	787 (72)	
Yes	1465 (22)	522 (27)	725 (20)	218 (20)	
Do not want to answer	217 (3)	43 (2)	84 (2)	90 (8)	
NA	0	0	0	0	
**Total floor area**					0.008
Very Small	1680 (25)	499 (26)	922 (25)	259 (24)	
Small	1676 (25)	450 (23)	986 (27)	240 (22)	
Large	1684 (25)	480 (25)	904 (24)	300 (28)	
Very Large	1681 (25)	505 (26)	891 (24)	285 (26)	
NA	32	15	6	11	
**Nature around home**					<0.001
Present	4893 (72)	1413 (72)	2749 (74)	731 (67)	
Absent	1860 (28)	536 (28)	960 (26)	364 (33)	
NA	0	0	0	0	
**Population density**					<0.001
Very Low	1686 (25)	563 (29)	888 (24)	235 (21)	
Low	1674 (25)	491 (25)	918 (25)	265 (24)	
High	1701 (25)	468 (24)	955 (26)	278 (25)	
Very High	1690 (25)	427 (22)	946 (26)	317 (29)	
NA	2	0	2	0	
**Wave**					0.208
Wave 2	3703 (55)	1041 (53)	2069 (56)	593 (54)	
Wave 3	3050 (45)	908 (47)	1640 (44)	502 (46)	
NA	0	0	0	0	

^1^Pearson's Chi-squared test

Overall, 29 % of respondents reported psychological distress, aligning with government and previous findings (29–31 %) (Ministry of Health Labour and Welfare, 2022; Sakurai et al., 2011). Among all respondents, 15 % held mortgages, with 26 % of these having ratios of ≥30 %, which was comparable to the 20 % rate found in Japan Housing Finance Agency (2023).

**Fig. 1:**
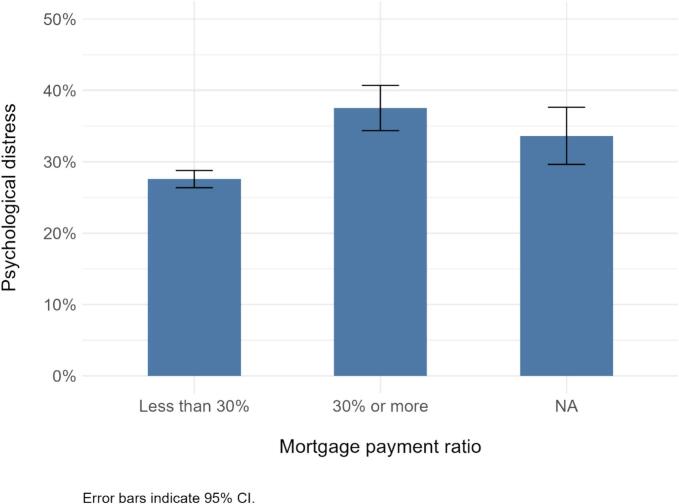
Prevalence of psychological distress by mortgage payment ratio in the Japan Housing and Health Cohort, 2023–2024.

Fig. 1 shows psychological distress prevalence by mortgage payment ratio using the dataset prior to multiple imputation. The prevalence was 28 % in the group with ratios <30 %, while it was 38 % in the group with ratios ≥30 %.

### 3.2 Two-level mortgage payment ratio and psychological distress

Using multiply imputed data (M = 20), Table 2 shows modified log Poisson regression results, with coefficients as PR. In the unadjusted model, homeowners with a mortgage payment ratio of ≥30 % compared with those with a ratio < 30 % had a PR of psychological distress of 1.35 (95 % CI 1.23–1.49) overall, 1.29 (95 % CI 1.15–1.45) for the high-income group and 1.51 (95 % CI 1.28–1.78) for the low-income group. After adjusting for covariates, for the overall sample, they were 1.22 times more likely to experience psychological distress (95 % CI 1.11–1.35). This adjusted association was significant in both high-income (PR = 1.23, 95 % CI 1.09–1.39) and low-income groups (PR = 1.21, 95 % CI 95 % CI 1.00–1.46), although the significance in the low-income group was marginal.

**Table 2:** Prevalence ratios for psychological distress comparing mortgage payment ratio ≥ 30 % with <30 % as reference overall and by income groups, in the Japan Housing and Health Cohort, 2023–2024.

		Crude^1^ PR (95 % CI)	Adjusted^2^ PR (95 % CI)
Overall	<30 %	1.00	1.00
	≥30 %	1.35 (1.23–1.49)	1.22 (1.11–1.35)
Low	<30 %	1.00	1.00
	≥30 %	1.51 (1.28–1.78)	1.21 (1.00–1.46)
High	<30 %	1.00	1.00
	≥30 %	1.29 (1.15–1.45)	1.23 (1.09–1.39)

Data presented as prevalence ratio (95 % CI) from modified log Poisson regression, with mortgage payment ratios <30 % as reference.

1. No control variables.

2. Models adjusted for age, gender, educational attainment, gross annual household income, household size, exercise habits, drinking habits, smoking status, significant life events in a year, total floor area, nature around home, population density, survey wave.

### 3.3 Six-level mortgage payment ratio and psychological distress

Table 3 shows the results of an analysis where the mortgage payment ratio variable used in Table 2 was recategorized from a binary variable into six categories (none, <10 %, approximately 10 %, 20 %, 30 %, and ≥ 40 %). Using multiply imputed data (M = 20) and the same controls shown in Table 2, each category served as a reference group, sequentially. The analysis revealed two key findings: First, high-income homeowners with a mortgage payment ratio of ≥40 % exhibited a significantly higher ratio of psychological distress. They were 1.74 times more likely to experience psychological distress than those without mortgages (95 % CI 1.38–2.19). The rates were also significantly higher compared to those with <10 % to 20 %. Additionally, they were 1.30 times more likely to experience distress than those with 30 % (95 % CI 1.03–1.64). In low-income groups, those with ≥40 % had no significant association with those with 10 %, 20 % and 30 %. Second, in high-income groups, no significant differences in psychological distress were found across groups with mortgage payment ratios of <10 %, 10 %, 20 % and 30 %. This suggests similar stress levels across low mortgage groups, while those with ≥40 % mortgage ratios or without mortgages showed different patterns.

**Table 3:** Adjusted prevalence ratios for psychological distress comparing mortgage payment ratio categories (none, <10 %, about 10 %, 20 %, 30 %, and ≥ 40 %) in high-income group, in the Japan Housing and Health Cohort, 2023–2024.

	Reference					
	None	<10 %	About 10 %	About 20 %	About 30 %	≥40 %
None	1.00	0.84 (0.69–1.01)	0.77 (0.64–0.91)	0.86 (0.74–1.00)	0.75 (0.63–0.89)	0.58 (0.46–0.73)
Less than 10 %	1.20 (0.99–1.45)	1.00	0.92 (0.75–1.12)	1.03 (0.86–1.23)	0.90 (0.73–1.10)	0.69 (0.53–0.89)
About 10 %	1.31 (1.09–1.56)	1.09 (0.89–1.33)	1.00	1.12 (0.96–1.31)	0.98 (0.82–1.17)	0.75 (0.60–0.95)
About 20 %	1.16 (1.00–1.35)	0.97 (0.81–1.16)	0.89 (0.76–1.04)	1.00	0.87 (0.75–1.01)	0.67 (0.54–0.83)
About 30 %	1.33 (1.12–1.59)	1.11 (0.91–1.36)	1.02 (0.85–1.22)	1.15 (0.99–1.33)	1.00	0.77 (0.61–0.97)
40 % or more	1.74 (1.38–2.19)	1.45 (1.12–1.87)	1.33 (1.05–1.68)	1.49 (1.21–1.85)	1.30 (1.03–1.64)	1.00

Data presented as prevalence ratio (95 % CI) from modified log Poisson regression. Reference category was set to none, <10 %, 10 %, 20 %, 30 %, and ≥ 40 %.

Models adjusted for age, gender, educational attainment, gross annual household income, household size, exercise habits, drinking habits, smoking status, significant life events in a year, total floor area, nature around home, population density, survey wave.

### 3.4 Sensitivity analysis: Homeowners under 65

Using multiply imputed data (M = 20), Table 4 shows the analysis of higher mortgage payment ratios and greater psychological distress for homeowners under 65 years old. In the unadjusted model, the PR of psychological distress was 1.24 (95 % CI 1.13–1.37) overall, 1.26 (95 % CI 1.05–1.50) for the low-income group, and 1.22 (95 % CI 1.08–1.37) for the high-income group. After adjustment, for the overall respondent group, compared with those with mortgage payment ratios <30 %, those with ≥30 % were 1.22 times more likely to experience psychological distress (95 % CI 1.10–1.35). This effect was also seen in the high-income group (PR = 1.23, 95 % CI 1.09–1.39), but was not statistically significant in the low-income group (PR = 1.19, 95 % CI 0.98–1.44).

**Table 4:** Prevalence ratios for psychological distress comparing mortgage payment ratio ≥ 30 % with <30 % as reference in homeowners aged under 65 years, in the Japan Housing and Health Cohort, 2023–2024.

		Crude^1^ PR (95 % CI)	Adjusted^2^ PR (95 % CI)
Overall	<30 %	1.00	1.00
	≥30 %	1.24 (1.13–1.37)	1.22 (1.10–1.35)
Low	<30 %	1.00	1.00
	≥30 %	1.26 (1.05–1.50)	1.19 (0.98–1.44)
High	<30 %	1.00	1.00
	≥30 %	1.22 (1.08–1.37)	1.23 (1.09–1.39)

Data presented as prevalence ratio (95 % CI) from modified log Poisson regression, with mortgage payment ratios <30 % as reference.

1. No control variables.

2. Models adjusted for age, gender, educational attainment, gross annual household income, household size, exercise habits, drinking habits, smoking status, significant life events in a year, total floor area, nature around home, population density, survey wave.

## 4 Discussion

This study, focusing on detached homeowners with above-average education and income in Japan, revealed that higher mortgage payment ratios were associated with greater psychological distress. The stratified analyses revealed the significant findings were limited to high-income households. Low-income results were marginally significant and not significant in sensitivity analysis.

Our results highlight two key aspects: 1) In comparison to prior literature that primarily focused on lower-income groups (Bentley et al., 2012; Bentley et al., 2016a; Baker et al., 2020a, Baker et al., 2020b; Arundel et al., 2024), this study suggests that high-income households may experience a similar relationship between high housing costs and psychological distress; 2) This relationship may be non-linear, with significant thresholds: one between the no-mortgage group and the low mortgage group (payment ratio of <10 %–30 %), and another between the low mortgage group and the high mortgage group (that of ≥40 %).

This study aligns with and expands on previous research linking housing costs to mental health. Cairney and Boyle (2004) and Bushman and Mehdipanah (2022) reported that higher housing cost burden was associated with greater psychological distress among homeowners with mortgages. Similarly, Mason et al. (2013) found a comparable association among homeowners in the top 50 % income bracket. While these earlier studies provided valuable insights, they involved specific limitations. The studies by Cairney and Boyle (2004) and Bushman and Mehdipanah (2022) did not conduct analyses by income level, whereas Mason et al. (2013) did not focus on high-income groups. The current study addresses these gaps in current knowledge, providing new insights into this relationship among high-income groups.

Conversely, our findings contrast with the results of some previous studies (Bentley et al., 2011; Park and Jung, 2019; Kuroki, 2023; Arundel et al., 2024). This may be because of differences in study conditions. Firstly, we focused specifically on the top 30 % of income earners and examined mortgage payment ratios, whereas an earlier study encompassed a broader income range such as the top 60 % and considered both rental and mortgage costs (Bentley et al., 2011). Secondly, the current study stratified homeowners by income levels, revealing nuanced relationships not seen when analyzing homeowners as a single group (Park and Jung, 2019; Kuroki, 2023; Arundel et al., 2024). Third, differences in outcome measurement methods may have influenced the discrepancy in results. In this study, we used the K6 scale to assess psychological distress, measuring respondents' condition over the past four weeks. In contrast, previous studies used the MCS to evaluate overall mental health over the past four weeks, and the CES-D to measure depressive symptoms during the previous week.

Two primary stress factors can be considered in the mechanism by which mortgage loans affect the psychological distress of homeowners. One is the manifest stress associated with debt, and the other is the latent stress unique to home ownership. Two studies by Drentea and Reynolds, 2012, Drentea and Reynolds, 2015 explored the relationship between debt and mental health. The first of these studies focused on the manifest stress associated with mortgage, arising from the obligation to repay borrowed money (Drentea and Reynolds, 2012). The second study further differentiated the effects of financial hardship and debt on mental health across income groups (Drentea and Reynolds, 2015). While financial hardship primarily affected low-income groups, debt had a more significant impact on the mental health of high-income individuals, who tend to borrow larger amounts proportional to their income. Therefore, these residents are more likely to experience significant stress associated with large payments. The second stress factor experienced by homeowners is latent stress. Nettleton and Burrows (2000) studied the psychological burden on homeowners who had difficulty making mortgage payments. The results showed that giving up owned housing and moving to cheaper rental housing was a significant mental stressor. Furthermore, Yilmazer et al. (2015) demonstrated an increase in the incidence of depression under such circumstances. Because housing is recognized as an indicator of social status, individuals who have difficulty repaying their mortgage may experience stigma as a stress factor because of a decrease in social evaluation (Gathergood, 2012). Results demonstrated that psychological distress levels significantly correlated with mortgage ratios, showing elevated distress in individuals with ratios ≥30 %, particularly those ≥40 %. This pattern suggests an association between higher mortgage payment ratios and greater latent psychological burden.

### 4.1 Limitations

This study had several limitations. First, we cannot completely rule out this association among low-income homeowners. The stratified analysis may have been underpowered to test the association among low-income households. Second, the generalizability of the study is limited, as it utilized a convenience sample restricted to high-income homeowners who purchased homes from a single housing manufacturer in Japan. This sampling approach could introduce selection bias, as participants were limited to customers of one specific company and those who voluntarily responded to the survey invitation. Third, despite excluding respondents with a history of mental health issues or sleep disorders, the cross-sectional design limits our ability to establish causal relationships between mortgages and psychological distress. Lastly, this study did not examine condominium owners or high-income renters, which could be areas for future research.

### 4.2 Policy implications

The percentage of households with a mortgage payment ratio ≥ 30 % has doubled from approximately 10 % in 2019 to 20 % in, 2023 (Japan Housing Finance Agency, 2023). This sharp increase could have significant impact on public mental health. However, public guidelines in Japan addressing the relationship between mortgage payment burden and mental health remain inadequately developed. As an initial measure, we propose that government agencies should implement awareness campaigns about the mental health risks associated with mortgage payment burden through websites and guidelines. Although high-income households may appear less vulnerable, they often delay seeking mental health treatment due to career disruption concerns, potentially leading to more severe conditions (Fukuda and Hiyoshi, 2012). Research findings suggest an association between mortgage payment burden and psychological impacts in this group, indicating potential value in including them in awareness initiatives.

## 5 Conclusion

The current findings revealed that higher mortgage payment ratios predict greater psychological distress even among high-income homeowners, extending previous findings on low-income groups and renters. These results suggest that housing cost stress is a broad public health concern.

## Funding

This work was supported by a grant from the Sekisui House Ltd., JSPS Grant-in-Aid for Research Activity Start-up (grant Number 24K22989) and the Project for Enhancing the Environment to Create Innovation in Regional Core Universities. The funders had no role in the interpretation, writing, or publication of this manuscript.

## CRediT authorship contribution statement

**Kazuya Ogawa:** Writing – original draft, Methodology, Formal analysis, Conceptualization. **Keiichi Shimatani:** Writing – review & editing, Methodology, Conceptualization. **Ryotaro Iwayama:** Data curation. **Norimichi Suzuki:** Writing – review & editing, Supervision.

## Declaration of competing interest

The authors declare the following financial interests/personal relationships which may be considered as potential competing interests: Kazuya Ogawa reports financial support was provided by JSPS Grant-in-Aid for Research Activity Start-up (grant Number 24K22989). Norimichi Suzuki reports financial support was provided by the Project for Enhancing the Environment to Create Innovation in Regional Core Universities. Keiichi Shimatani reports financial support was provided by Sekisui House, Ltd. Ryotaro Iwayama reports a relationship with Sekisui House, Ltd. that includes: employment. Ryotaro Iwayama is concurrently pursuing graduate studies at Chiba University. If there are other authors, they declare that they have no known competing financial interests or personal relationships that could have appeared to influence the work reported in this paper.

## Data Availability

The authors do not have permission to share data.
